# The results of a survey highlighting issues with feedback on medical training in the United Kingdom and how a Smartphone App could provide a solution

**DOI:** 10.1186/s13104-015-1649-z

**Published:** 2015-11-06

**Authors:** Thomas G. Gray, Gill Hood, Tom Farrell

**Affiliations:** Faculty of Health and Wellbeing, Sheffield Hallam University, City Campus, Howard Street, Sheffield, S1 1WB UK; Department of Postgraduate Medical Education, Sheffield Teaching Hospitals NHS Foundation Trust, Royal Hallamshire Hospital, Glossop Road, Sheffield, S10 2JF UK

**Keywords:** Smartphone, App, Feedback, Medical education

## Abstract

**Background:**

Feedback drives learning in medical education. Healthcare Supervision Logbook (HSL) is a Smartphone App developed at Sheffield Teaching Hospitals for providing feedback on medical training, from both a trainee’s and a supervisor’s perspective. In order to establish a mandate for the role of HSL in clinical practice, a large survey was carried out.

**Methods:**

Two surveys (one for doctors undertaking specialty training and a second for consultants supervising their training) were designed. The survey for doctors-in-training was distributed to all specialty trainees in the South and West localities of the Health Education Yorkshire and the Humber UK region. The survey for supervisors was distributed to all consultants involved in educational and clinical supervision of specialty trainees at Sheffield Teaching Hospitals.

**Results:**

The results confirm that specialty trainees provide feedback on their training infrequently—66 % do so only annually. 96 % of the specialty trainees owned a Smartphone and 45 % said that they would be willing to use a Smartphone App to provide daily feedback on the clinical and educational supervision they receive. Consultant supervisors do not receive regular feedback on the educational and clinical supervision they provide to trainees—56 % said they never received such feedback and 33 % said it was only on an annual basis. 86 % of consultants surveyed owned a Smartphone and 41 % said they would be willing to use a Smartphone App to provide feedback on the performance of trainees they were supervising.

**Conclusions:**

Feedback on medical training is recorded by specialty trainees infrequently and consultants providing educational and clinical supervision often do not receive any feedback on their performance in this area. HSL is a simple, quick and efficient way to collect and collate feedback on medical training to improve this situation. Good support and education needs to be provided when implementing this new technology.

## Background

Good quality feedback on performance is essential for driving learning and development for doctors-in-training [1] and likewise to help supervisors who are providing educational or clinical supervision to doctors-in- training to develop as trainers and leaders.

Educational supervision for doctors-in-training is currently provided by a nominated educational supervisor, usually a consultant, for each trainee [2]. The educational supervisor meets with the doctor-in-training at least three times a year and sets educational goals and reviews completed work-based assessments. Usually doctors-in-training are required to complete a specific number of work-based assessments (WBAs) each year, which assess clinical skills (history, examination, management decisions etc.), professional skills (such as team working, clinical judgment and communication skills) and practical skills (such as operations performed under supervision or clinical skills such as venipuncture). The results of these assessments are used to inform the educational goals set by the educational supervisor and the doctor-in-training. Day-to-day ‘clinical supervision’ is then provided to the doctor-in-training as they undertake their role as a doctor delivering a service [2]. Not all of the time working by a doctor-in-training will be spent receiving specific training, rather some of that time will be spent in service provision—usually treating and managing patients with no direct upward clinical supervision. Often the amount of training received is adversely affected due to service pressures associated with medical staff shortages in the UK [3]. This is due in part to reductions in the number of training posts and the impact of the European Working Time Regulations on hours of work. This has impacted on the amount and quality of training for doctors in the UK [4].

Currently, doctors in the UK undertaking specialty training provide feedback on the training they receive on an annual basis [5]. Mechanisms for this include the GMC National Training Survey [6] and local Health Education England (HEE) regional surveys. By the time that feedback from these mechanisms is collated and analysed the doctors-in-training who have provided the feedback have often rotated to a different post and will therefore not benefit from changes implemented as a result of the feedback they provide [7].

At the present time there is currently no clear national mechanism for doctors in substantive posts (consultants) providing educational and clinical supervision to receive specific feedback about the quality of the supervision they provide.

In order to assess opinions and establish perceptions amongst doctors-in-training and their consultant supervisors with regard to how feedback on medical training is currently collected and utilised a large survey was carried out.

This paper will present and analyse the results of the survey and discuss how using *Healthcare Supervision Logbook* Smartphone App could form a solution to the issues highlighted.

## Methods

This study was registered as a service evaluation at the clinical effectiveness unit at Sheffield Teaching Hospitals and ethical approval was not required. Two separate surveys (one for doctors undertaking specialty training and a second for consultants supervising their training) were designed. The surveys were distributed, managed and analysed using a recognised survey website system (Surveymonkey.com) [8].

The survey for doctors-in-training was distributed to all specialty trainees in the South and West localities of the Health Education Yorkshire and the Humber region (n = 3026). The survey for supervisors was distributed to all consultants involved in educational and clinical supervision of specialty trainees at Sheffield Teaching Hospitals (n = 764).

The survey for doctors in training comprised six short questions (see Table 1), all of the answers were multiple choice:

**Table 1:** Survey questions for doctors-in-training

1.	Which year group are you in?
2.	How frequently are you currently able to PROVIDE feedback about the clinical and educational supervision you receive as part of your training?
3.	Do you feel that you get enough opportunities to PROVIDE feedback on the clinical and educational supervision you receive as a specialty trainee?
4.	Do you feel that the feedback you RECEIVE on your performance as a specialty trainee is representative of your abilities?
5.	Do you own a Smartphone (e.g. Apple iPhone, Samsung Galaxy)?
6.	If yes, would you be willing to use an App on your Smartphone to provide daily feedback on the educational and clinical supervision you receive?

The survey for consultant supervisors comprised eight short questions along similar lines (see Table 2).

**Table 2:** Survey questions for consultant supervisors

1.	How long have you been involved in educational or clinical supervision?
2.	How frequently are you able to PROVIDE formal recorded feedback about a trainee’s performance?
3.	Do you feel that you get enough opportunities to PROVIDE accurate feedback on a trainee’s performance?
4.	What in your opinion prevents you from providing regular feedback about the trainees you are supervising?
5.	How frequently do you currently RECEIVE formal feedback on your educational or clinical supervision activities from trainees?
6.	Do you feel that you RECEIVE enough feedback on your performance as an educational or clinical supervisor for trainees?
7.	Do you own a Smartphone (e.g. Apple iPhone, Samsung Galaxy)?
8.	If yes, would you be willing to use an App on your Smartphone to provide daily feedback on the performance of the trainee you are supervising after each clinical session?

The surveys were each live for a period of 4 weeks, during which time a weekly reminder was sent to all potential participants by email.

The results were analysed using the Survey Monkey content management system [8] and Microsoft Excel.

## Results and discussion

499 (16.4 %) trainees and 154 consultant supervisors (20.2 %) completed the respective surveys. These response rates are similar to and actually slightly higher than previous similar published studies [9, 10] and represented a large number of doctors’ views. Attempts were made to maximize the response rate- with four reminders sent out by email. Without making the questionnaire mandatory for trainees and supervisors to complete it would be difficult to increase the response rate further.

Of those trainees who completed the trainee survey, 262 (52.6 %) were in year one to three of their training (ST1-ST3), 140 (28.1 %) were in the fourth to sixth year of their training (ST4-ST6) and 96 (19.3 %) were in the seventh year of their training or beyond (ST7+). With regard to the consultant supervisors, 21 had been involved with educational or clinical supervision at this level for 1–3 years (13.6 %), 30 for 4–6 years (19.5 %) and 103 for 6 years plus (66.9 %).

### Specialty trainees

When asked how frequently they were currently able to provide formal feedback about the quality of the educational and clinical supervision they received as part of their training, 65.6 % said annually and 21 % said monthly, 13.4 % thought that it was more frequently than this. See summary of results in Table 3.

**Table 3:** Summary of results for specialty trainees

Question	Summary of responses
Which ST (specialty trainee) year group are you in?	ST1–ST3: 52.6 % ST4–ST6: 28 % ST7+: 19.3 %
How frequently are you currently able to PROVIDE feedback about the clinical and educational supervision you receive as part of your training?	Annually: 65.6 % Monthly: 21 % Fortnightly or more frequently: 13.4 %
Do you feel that you get enough opportunities to PROVIDE feedback on the clinical and educational supervision you receive as a specialty trainee?	Strongly disagree: 8.6 % Disagree: 33.6 % Neither agree nor disagree: 26.6 % Agree: 26.8 % Strongly agree: 4.4 %
Do you feel that the feedback you RECEIVE on your performance as a specialty trainee is representative of your abilities?	Strongly disagree: 1.6 % Disagree: 12.8 % Neither agree nor disagree: 24.8 % Agree: 55.6 % Strongly agree: 5.2 %
Do you own a Smartphone (e.g. Apple iPhone, Samsung Galaxy)?	Yes: 95.8 % No: 4 % Not sure: 0.2 %
If yes, would you be willing to use an App on your Smartphone to provide daily feedback on the educational and clinical supervision you receive?	Yes (overall): 48.8 % Yes (ST1–3): 52 % yes Yes (ST4–6): 41 % yes Yes (ST7+): 40.4 % yes

Asked if they felt that they had enough opportunities to provide feedback on the educational and clinical supervision received as part of their training, 42.2 % disagreed or strongly disagreed compared with 34.6 % who agreed or strongly agreed with this.

Specialty trainees were asked if they felt that feedback they received on their performance was representative of their abilities; 60.8 % agreed or strongly agreed with this statement, whilst 14.4 % disagreed or strongly disagreed and 24.8 % were unsure.

95.8 % of the specialty trainees said that they owned a Smartphone and 44.8 % said that they would be willing to use a Smartphone App to provide daily feedback on the clinical and educational supervision they receive. 24.8 % were unsure about using a Smartphone App for this purpose and 30.4 % said they were unwilling when completing the survey.

### Consultant supervisors

Consultant supervisors were asked how frequently they were able to provide documented feedback about a trainee’s performance. 5.8 % said annually, 40.9 % monthly, 16.8 % fortnightly, 29.8 % weekly and 5.8 % daily. See summary of results in Table 4.

**Table 4:** Summary of results for consultant supervisors

Question	Summary of responses
How long have you been involved in educational or clinical supervision?	1–3 years: 13.6 % 4–6 years: 19.5 % 7 years+: 66.9 %
How frequently are you able to PROVIDE formal recorded feedback about a trainee’s performance?	Annually: 5.8 % Monthly: 40.9 % Fortnightly: 16.8 % Weekly: 29.8 % Daily: 5.8 %
Do you feel that you get enough opportunities to PROVIDE accurate feedback on a trainee’s performance?	Strongly disagree: 4.55 % Disagree: 39.6 % Neither agree nor disagree: 23.4 % Agree: 26 % Strongly agree: 6.5 %
What in your opinion prevents you from providing regular feedback about the trainees you are supervising?	Lack of time: 50.7 % Lack of clear mechanisms to do so: 16.6 % Lack of access to computers: 8.5 % Not a priority: 4.2 % Other: 20 %
How frequently do you currently RECEIVE formal feedback on your educational or clinical supervision activities from trainees?	Never: 55.8 % Annually: 33.1 % Monthly: 8.4 % Weekly: 0.7 % Daily: 0 %
Do you feel that you RECEIVE enough feedback on your performance as an educational or clinical supervisor for trainees?	Strongly disagree: 18.8 % Disagree: 46.8 % Neither agree nor disagree: 22.1 % Agree: 9.7 % Strongly agree: 2.6 %
Do you own a Smartphone (e.g. Apple iPhone, Samsung Galaxy)?	Yes: 85.7 % No: 13 % Not sure: 1.3 %
Would you be willing to use an App on your Smartphone to provide daily feedback on the educational and clinical supervision you receive?	Yes: 40.9.2 % No: 31.2 % Unsure: 27.9 %

In response to whether they felt that they had enough opportunities to provide feedback about the performance of specialty trainees, 32.5 % disagreed or strongly disagreed with this, 23.4 % were unsure, whilst 44.2 % agreed or strongly agreed that they had enough opportunities to provide feedback.

When asked what they felt prevented them from providing more regular feedback about specialty trainee’s performances, 50.7 % cited a lack of time. 16.6 % said it was due to a lack of clear mechanisms to do so, 8.5 % felt that it was because of poor access to computers and 4.2 % said that it was because it was not a priority for them.

The consultant supervisors were also asked how frequently they received formal feedback about their educational or clinical supervision activities from trainees: 55.8 % said they never received such feedback, whilst 33.1 % said it was on an annual basis.

Asked if they felt that they received enough feedback about their performance as an educational or clinical supervisor for specialty trainees, 65.6 % disagreed or strongly disagreed with this, whilst 22.1 % were unsure and 12.3 % agreed.

85.7 % of consultants surveyed owned a Smartphone and 40.9 % said they would be willing to use a Smartphone App to provide feedback on the performance of trainees they were supervising, 31.2 % said that they would be unwilling to do this at the time of the survey and 27.9 % were unsure about this.

The survey results help to highlight a number of problems with the current pattern for provision of feedback on medical training. It is clear that the majority of trainees (66 %) are only able to provide feedback on their training on an annual basis and that more than two-thirds of trainees (69 %) do not agree that they are able to provide feedback frequently enough.

The feedback provided by specialty trainees is often not reflective of the type of training environment or of individual supervisors providing educational or clinical supervision. This is because feedback is usually generic regarding the training placement as a whole, including different training environments (clinics, theatres, on call etc.) and educational and clinical supervision from a number of different supervisors.

The majority of consultant supervisors feel that they never receive formal feedback on their educational or clinical supervision activities from trainees and only 12 % of those questioned felt that there was currently enough feedback from trainees to supervisors.

Using Smartphone App technology could represent a solution to these problems.

### Healthcare Supervision Logbook

**Fig. 1:**
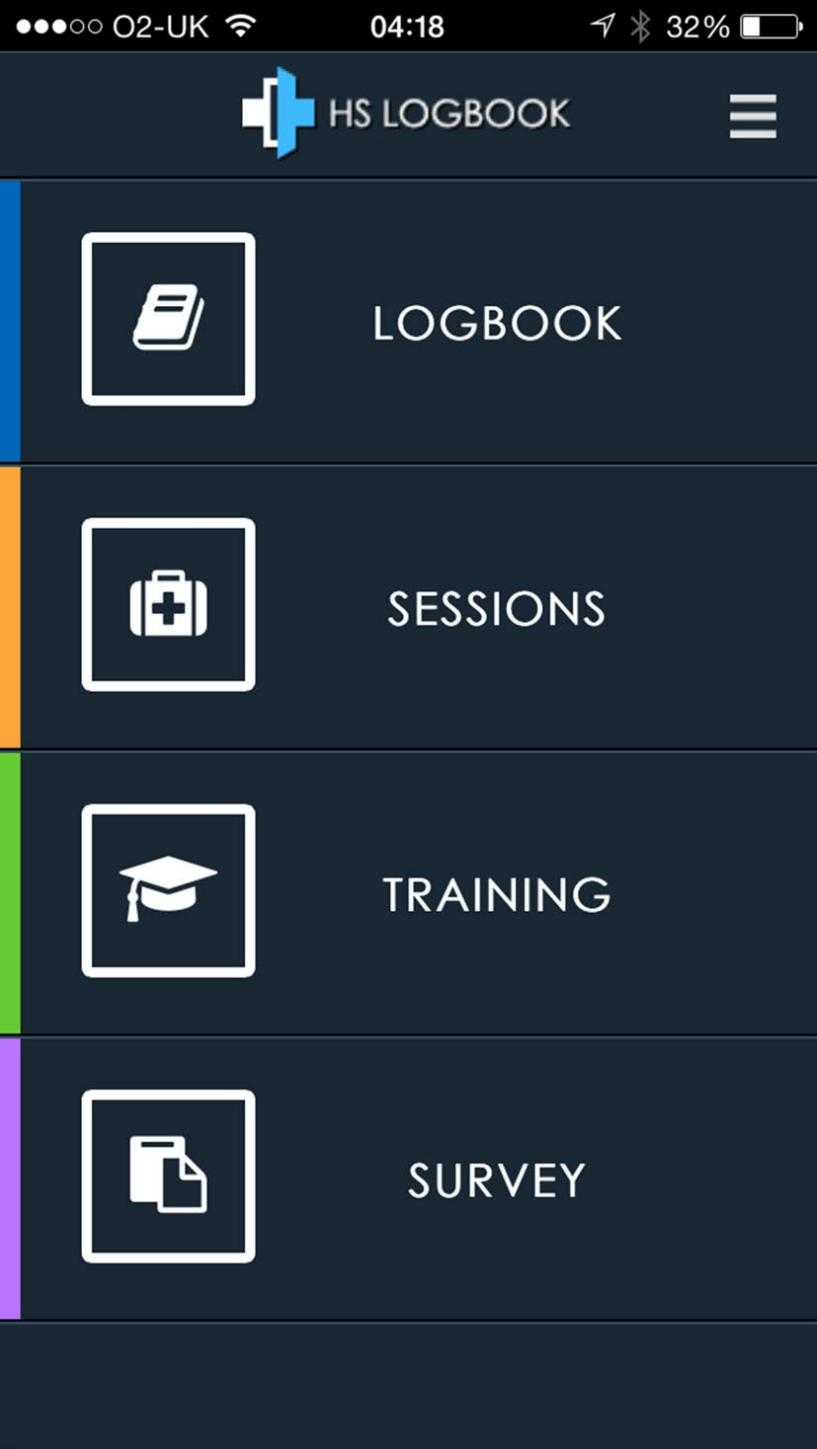
A screen shot of the main menu of the supervisor’s version of *Healthcare Supervision Logbook*

A Smartphone App for providing feedback on medical training from both a trainee’s and supervisor’s perspective has been developed at Sheffield Teaching Hospitals with the support of Health Education England Yorkshire and Humber [7]. It is called *Healthcare Supervision Logbook*. It can be used by both doctors-in-training and their supervisors to provide feedback on medical training and therefore is available in both a trainee and a supervisor version. *Healthcare Supervision Logbook* has three functions common to both trainee and supervisor versions: ‘Sessions’, ‘Logbook’ and ‘Survey’. The supervisor version also has a ‘Training’ function (Fig. 1).

This App benefits trainees by providing a platform for them to rate the educational value of a clinical or teaching session they attend, including the educational and clinical supervision provided in the session (‘Sessions’ function). This function allows sessions within a department to be rated on a ten-point scale for training quality; therefore allowing training program organisers to appropriately assess the standard of training provided within a specific department or departmental area. This information can be used to identify trends in training provision and can be used to provide educational and clinical supervisors with feedback on their skills as such.

This function can be anonymised so that the rating is provided for a department rather than an individual supervisor. It is possible to add the names of specific supervisors, so that feedback specific to an individual supervisor rather than a department could be recorded. This function could benefit the supervisors by providing them with specific individual feedback, which will allow them to reflect on the training they provide and develop their skills in this area. The data generated from this ‘Sessions’ feedback function will help to assess clinical placements and assist in allocation of trainees to the unit likely to provide them with the best training opportunities. Supervisors can benefit from using a pared down version of the ‘Sessions’ function within their version of *Healthcare Supervision Logbook* to record their clinical activities for purposes of appraisal and revalidation.

The supervisors’ version of *Healthcare Supervision Logbook* provides a platform for the educational or clinical supervisor to record an assessment of the trainee’s performance after each session in which they have supervised a trainee (‘Training’ function).

This function will benefit trainees by collecting multisource feedback after every clinical session [11]. These data can be used to create a report on a trainee’s performance on a regular basis, from a number of different supervisors in a number of different training environments. This will provide valuable information, highlighting training needs and areas for trainee improvement and development far more promptly than current mechanisms of feedback allow. *Healthcare Supervision Logbook’s* ‘Training’ function also benefits supervisors by providing a way for them to collect evidence of specific involvement in educational and clinical supervision, which they can use to support their continuous professional development, appraisals and revalidation.

**Fig. 2:**
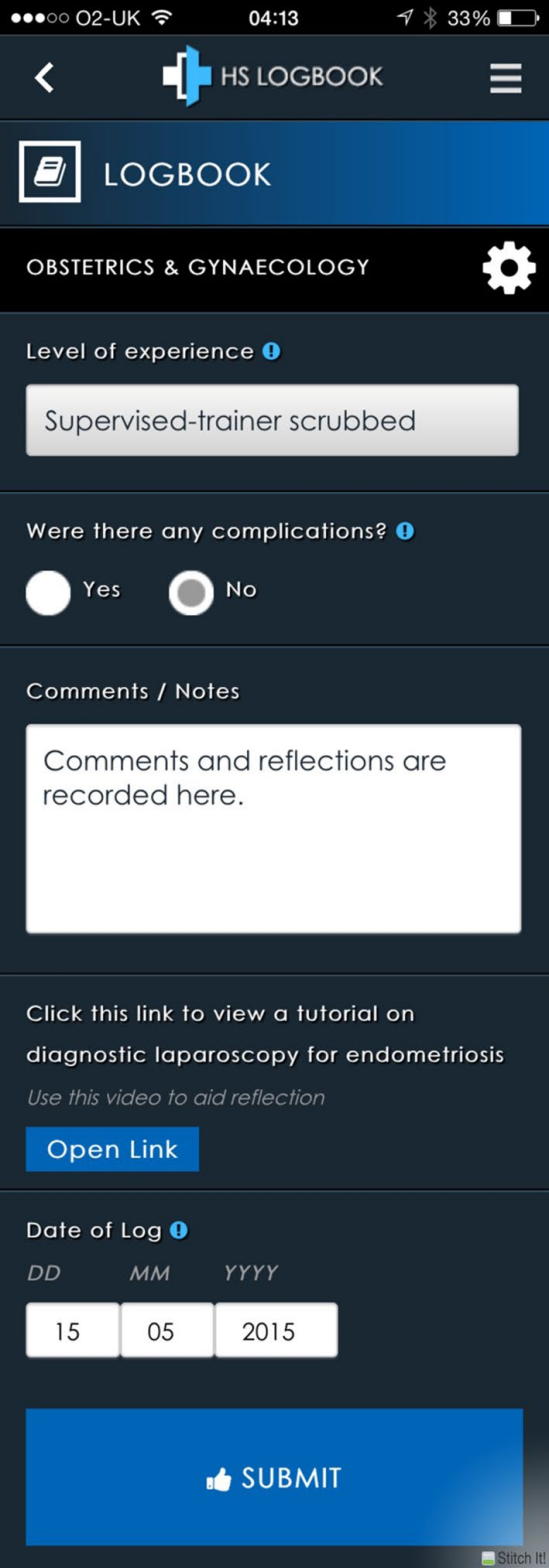
An extended screenshot of the ‘logbook’ function of *Healthcare Supervision Logbook*

*Healthcare Supervision Logbook* also has mechanisms allowing for recording of practical procedures performed using a specialty-specific logbook (‘Logbook’ function) and has mechanisms for collecting feedback from patients using a GMC- approved form [12] (‘Survey’ function). Within the logbook function there is a mechanism for attaching, to each specific skill, a link to a video or guideline/paper to aid reflection (Fig. 2).

The content of *Healthcare Supervision Logbook* is fully modifiable, allowing for questions to be modified and added and for adaptation to multiple specialties and disciplines [7].

### A role for *Healthcare Supervision Logbook* ‘Sessions’ function

The main benefits of the ‘Sessions’ function for trainees is that it allows them to provide meaningful feedback on the training they are receiving after every training session they attend, not just on an annual or placement-by-placement basis.

Regular completion of the ‘Sessions’ function will enable the trainee to keep a ‘training record’ of the sessions they have attended, for purposes of their annual review.

From the supervisor’s perspective, trainees completing the ‘Sessions’ feedback will enable the department/supervisor to obtain evidence of the quality of training perceived by the trainees. This function could obviously play a major role in the quality assurance process for medical training within the department.

This survey demonstrated a clear deficiency in the frequency with which consultants receive feedback about the educational and clinical supervision they provide to trainees—56 % said that they never received such feedback. At the present time there are very few mechanisms to provide feedback on training experiences. The previously mentioned GMC National Training Survey [6] and Health Education England Regional surveys are usually the only mechanisms for this which exist—presently these occur on an annual basis and the results are not usually available until the specialty trainees have rotated to a different placement. One problem with annual feedback is that trainees may over-focus on negative aspects of their training, as this is the only formal opportunity they have to express their viewpoint. Similarly, there is no opportunity to rate supervisors as individuals as this information is collected on a departmental basis.

**Fig. 3:**
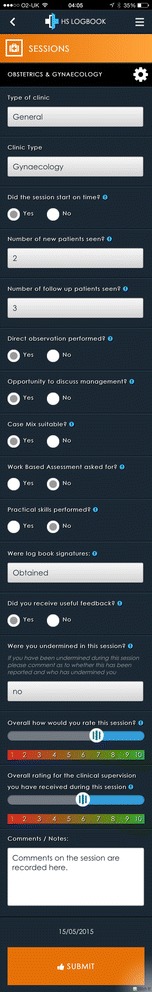
An extended screenshot of the ‘sessions’ function of *Healthcare Supervision Logbook*

An example of the information obtained by a trainee completing a ‘Sessions’ feedback log for a theatre session is as follows (Fig. 3). The session feedback log will ask the trainee about whether the session started and ended on time, the number of minor and major cases they have performed, whether the case mix was appropriate, whether they had the opportunity to operate with supervision, whether the clinical supervision they received was adequate or not, if they were able to see the patients postoperatively, whether work-based assessments were asked for and whether any undermining occurred during the theatre session.

Using the *Healthcare Supervision Logbook* App to provide feedback on a session takes approximately 50 s. As it is available on the users personal device, does not require Internet access and is rapid to use, it facilitates recording of meaningful feedback with ease.

### A role for *Healthcare Supervision Logbook* ‘Training’ function

The supervisor would complete a ‘Training’ entry about the trainee’s performance at the end of each training session. For the supervisor this provides evidence of frequent involvement in educational and clinical supervision, collecting evidence for appraisal and revalidation. From the trainee’s perspective this will provide enhanced 360° multisource feedback, which can be provided to the trainee on a quarterly (or more frequently if required) basis to facilitate educational progress meetings and annual appraisal.

The survey showed that 60 % of specialty trainees felt that the feedback they received was representative of their abilities, which is reassuring. However, 40 % were unsure or disagreed with this, which shows an area for development. The enhanced 360° multisource feedback provided by the ‘Training’ function would help to create a more accurate picture of a trainee’s performance over time with assessments by multiple assessors, in multiple training environments, supervising the trainee. It is anticipated that continuous assessment of this sort is likely provide a more representative picture of a trainee’s performance compared to infrequent or ‘one-off’ assessments.

**Fig. 4:**
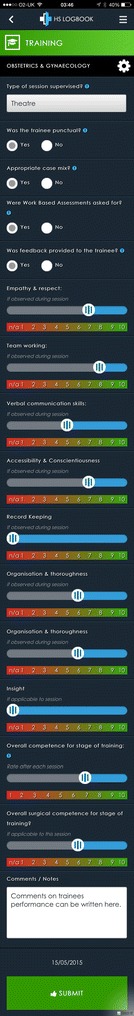
An extended screenshot of the ‘training’ function of *Healthcare Supervision Logbook*

By using *Healthcare Supervision Logbook’s* ‘Training’ function (Fig. 4) to provide feedback, supervisors from all specialties could realistically provide daily feedback in a way that would be minimally time consuming (under 1 min) and avoid the need to access computers as well as providing a clear mechanism for doing so. These were the main reasons for not providing more regular feedback highlighted by the supervisor survey.

An example of the data generated by the ‘Training’ component of *Healthcare Supervision Logbook* is as follows. The type of session is selected and then the supervisor answers four yes or no questions regarding punctuality, whether the case mix was appropriate for the trainee, whether work-based assessments were asked for and whether feedback was provided to the trainee. The Supervisor then rates the trainee using a ten-point visual analogue slider for eight areas relevant to training. These are: empathy and respect, team-working, verbal communication skills, accessibility and conscientiousness, clinical judgment, record keeping, organisation and thoroughness and insight. Not all of these areas will be applicable to each training session. The consultant supervisor is then asked to rate the trainee for their overall competence for their stage of training and, if relevant, their overall surgical competence for their stage of training. By using the training component of *Healthcare Supervision Logbook* to provide feedback about a trainee’s performance after every session, a far more accurate picture is built up of how a trainee is performing. The content management system allows each trainee to be compared to others in their department and beneficial summary reports can be generated which allow for easy assessment of each trainee, allowing for this function to support accurate appraisal. Again, as it is a function within a Smartphone App this is simple and quick to use and does not rely on Internet access, computer availability or the trainee remembering to ask.

### Smartphone ownership and acceptability of *Healthcare Supervision Logbook*

Smartphone ownership amongst trainees is high at 98 %+, this is higher than in previous publications which have demonstrated Smartphone ownership amongst doctors to be 74.8 % [9] and 85 % [10] in 2012. This is an expected trend as mobile technology continues to become increasingly integrated into daily life. There is evidence that up to 75 % of junior doctors already use medical Apps on their Smartphones in the workplace [13] and a vast number of medical Apps are available for purchase [14, 15], many for free or at a minimal cost. Acceptability of the use of Smartphones and medical Apps in the workplace is high [10, 13]. When asked about willingness to use a Smartphone App to provide feedback on training, the majority of specialty trainees (44.8 %) were willing to do so. However 28.2 % said that they were unwilling, which was a larger number than expected. Issues with the use of medical Apps cited in previous studies has included their cost, the lack of useable content and concerns about the appearance of using a mobile phone in the workplace [9]. Equally, there are many time pressures on junior doctors and there may have been concerns about the extra time needed to use an App to provide feedback on training, as well as skepticism about the potential effect of such feedback in effecting change. There may also have been concerns about confidentiality of data recorded- trainee’s may prefer to provide feedback anonymously for fear of negative repercussions if they provide poor feedback about their training. The security of data recorded may also have been a concern.

When the results of the trainee survey are split by specialty trainee year group—those in ST1-3 were far more willing to use a Smartphone App for providing feedback on training (52 %) compared with those in more senior years (41 % for ST4-6 and 40.4 % for ST6+). This could be due to increased familiarity with Smartphone technology and Apps in younger years, or increased cynicism about the provision of feedback on training by those in older years. It is clear that concerns about using a Smartphone App to provide feedback will need to be listened to carefully. With clear education about the functions and uses of *Healthcare Supervision Logbook* to provide feedback on day-to-day training experiences, the authors hope that the number of junior doctors willing to use this Smartphone App will increase.

The survey showed that ownership of Smartphones amongst consultant staff was also high-86 %. Overall, 40.9 % said they would be willing to use a Smartphone App to assess a trainee’s performance on a daily basis. Younger consultants were more willing to use Healthcare supervision Logbook, with 42 % of those involved in supervision for 1–3 years willing to use the App. Hopefully, with good planning, communication, information and support many of these consultant supervisors would become willing to use a well-designed Smartphone App to provide feedback on a trainee’s performance- particularly as they would be able to use *Healthcare Supervision Logbook* to record the practical procedures that they perform, collect data on their sessions for purposes of appraisal and revalidation and provide evidence of training and teaching which would also support revalidation.

## Conclusions

This study helps to establish current perceptions with regard to feedback on medical training from both specialty trainee’s and consultant supervisor’s perspectives. The data generated helps to provide evidence for the use of different mechanisms for collecting and providing feedback on medical training in the UK, compared to current methods.

It is clear that feedback on training is provided infrequently, both with regard to the performance of specialty trainees who are being trained and perceived quality of the training provided to specialty trainees by consultant supervisors. Use of Smartphone technology, using a well-designed and simple-to-use Smartphone App such as *Healthcare Supervision Logbook* to provide daily session-by-session feedback on training experiences from both a specialty trainee’s and a consultant trainer’s perspective would solve many of the problems highlighted by this survey, including the frequency with which feedback is recorded and issues regarding time constraints and computer access. Introducing *Healthcare Supervision Logbook* into everyday clinical practice will require excellent communication and support. *Healthcare Supervision Logbook* has the potential to revolutionise the way feedback on training is provided, improving training for doctors and supporting excellent healthcare provision for all [16].

## References

[1] Gordon J (2003). One to one teaching and feedback. BMJ.

[2] The General Medical Council. Roles and Responsibilities of an educational supervisor. http://www.gmc-uk.org/Final_Appendix_2___Roles_of_Supervisors.pdf_53817452.pdf. Accessed 29 Mar 2015.

[3] Geraint Fuller, Simpson Iain A (2014). “Modernising Medical Careers” to “Shape of Training”—how soon we forget. BMJ.

[4] Royal College of Surgeons of England, Work Time Directive Taskforce report. https://www.rcseng.ac.uk/policy/documents/wtd-taskforce-report-2014. Accessed 19 Sep 2014.

[5] The General Medical Council. The Trainee Doctor. http://www.gmc-uk.org/Trainee_Doctor.pdf_39274940.pdf. Accessed 29 Mar 2015.

[6] The General Medical Council. The National Training Survey. http://www.gmc-uk.org/education/surveys.asp. Accessed 29 Mar 2015.

[7] Gray T, Hood G, Farrell T (2014). The development and production of a novel Smartphone App to collect day-to-day feedback from doctors in training and their trainers. BMJ Innov.

[8] Survey Monkey. https://www.surveymonkey.com. Accessed 29 Mar 2015.

[9] Payne K, Wharrad H, Watts K (2012). Smartphone and medical related App use among medical students and junior doctors in the United Kingdom (UK): a regional survey. BMC Med Inform Decis Mak.

[10] Franko O, Tirrell T (2012). Smartphone App use among medical providers in ACGME training programs. J Med Syst.

[11] Violato C, Lockyer JM, Fidler H (2003). Multisource feedback: a method of assessing surgical practice. BMJ.

[12] General Medical Council. GMC questionnaires. http://www.gmc-uk.org/doctors/revalidation/colleague_patient_feedback_resources.asp. Accessed 29 March 2015.

[13] Ozdalga E, Ozdalga A, Ahuja N (2012). The Smartphone in medicine: a review of current and potential use among physicians and students. J Med Internet Res..

[14] Mosa A, Yoo I, Sheets L (2012). A systematic review of healthcare applications for Smartphones. BMC Med Inform Decis Mak.

[15] Derbyshire E, Dancey D. Smartphone medical applications for women’s health: what is the evidence-base and feedback? International Journal of Telemedicine and Applications 2013. http://www.hindawi.com/journals/ijta/2013/782074/. Accessed 19 Sep 2014. 10.1155/2013/782074 PMC3880694 24454354

[16] UK Department of Health. High quality care for all 2008. http://webarchive.nationalarchives.gov.uk/+/www.dh.gov.uk/en/healthcare/highqualitycareforall/index.htm. Accessed 19 Mar 2014.

